# Assessing the Consultation Pattern from Emergency Room Physicians to General Surgery Subspecialties: Identifying the Most Frequently Consulted Subspecialty

**DOI:** 10.3390/healthcare13222955

**Published:** 2025-11-18

**Authors:** Ibrahim Tawfiq Al Babtain, Wed Khalid Alwabel, Bader Abdulhadi Alhoumaily, Nawaf Abdullah Alqahtani, Renad Mousa Almasari, Hashim Tariq Tatwani

**Affiliations:** 1 Department of Surgery, The Ministry of National Guard Hospital, Riyadh 22490, Saudi Arabia; 2 College of Medicine, King Saud Bin Abdulaziz University for Health Sciences, Riyadh 3660, Saudi Arabiahashim1212@outlook.com (H.T.T.)

**Keywords:** acute care, ED, surgical complaints procedures

## Abstract

**Background:** The acute care surgery (ACS) model employs a 24/7 multidisciplinary team—surgeons, nurses, and residents—supported by an electronic consultation system to optimize emergency The acute care surgery (ACS) model provides 24/7 multidisciplinary management of emergency surgical patients. This study aimed to describe the demographic and clinical characteristics of patients admitted from the emergency department (ED) under general surgery, identify the most common presenting complaints and operative procedures, and determine which general surgery subspecialties were most frequently consulted at King Abdulaziz Medical City (KAMC). **Methods:** We conducted a retrospective study at KAMC, Riyadh (MNGHA), from September 2022 to November 2023. A total of 384 ED patients admitted under general surgery were included. Data were extracted from the BestCare electronic medical record and analyzed for demographics, presenting complaints, operative procedures, and subspecialty consultations. **Results:** Of 384 patients, 204 (53.1%) were male and 180 (46.9%) were female. The largest age group was 30–45 years (*n* = 112, 29.2%), followed by <30 years (*n* = 98, 25.5%). Leading presenting complaints were abdominal pain (*n* = 243, 63.3%), fever with nausea/vomiting (*n* = 68, 17.7%), and rectal pain/bleeding (*n* = 44, 11.5%). Laparoscopic cholecystectomy was the most common procedure (*n* = 123, 32.0%), followed by laparoscopic appendectomy (*n* = 57, 14.8%). ACS received most consultations (*n* = 231, 61.8%), with additional referrals to colorectal surgery (*n* = 86, 23.0%) and upper gastrointestinal surgery (*n* = 40, 10.7%). Nearly all consult requests originated in the ED (*n* = 355, 98.9%). **Conclusions:** Abdominal pain was the predominant ED complaint prompting surgical referral, and laparoscopic cholecystectomy and appendectomy were the most frequently performed procedures. ACS was the primary subspecialty consulted, underscoring its central role in emergency surgical care at KAMC.

## 1. Introduction

At King Abdulaziz Medical City (KAMC), acute care surgery (ACS) and emergency general surgery (EGS) are related but distinct terms. In local practice, ACS refers to a service model that integrates emergency general surgery, trauma surgery, and surgical critical care, whereas EGS specifically pertains to the urgent operative and non-operative management of non-trauma general surgical conditions. Referrals from the emergency department (ED) are initially directed to the on-call ACS team, which triages and manages cases and engages subspecialty services—such as colorectal, upper gastrointestinal, or hepatobiliary surgery—when advanced expertise is required. This structure ensures timely care while allowing subspecialists to be consulted for complex anatomy, technically demanding procedures, or situations where specialized operative techniques are necessary to optimize outcomes [1,2].

The electronic consultation system, known as the BestCare System at King Abdulaziz Medical City (KAMC), was developed to facilitate communication and coordination among physicians across various specialties and plays a crucial role in optimizing patient care and facilitating favorable clinical outcomes. Effective use of electronic consultations has been associated with reduced consultation times, enhanced diagnostic accuracy and clinical management, and improved patient satisfaction. All of these factors play an especially critical role in acute care surgery and emergency department settings [3,4]. The consultation rate and consultation-to-decision time is defined as the interval from when a specialty service accepts a consultation to when a disposition decision is made. These are crucial metrics influencing efficiency and patient flow through the ED. These are key determinants of emergency care performance, as any delays in this process can significantly increase ED wait times, thereby negatively impacting patient care [5,6].

Emergency General Surgery (EGS) is one of the primary specialties consulted by emergency departments for patients requiring urgent surgical intervention [7,8]. Common conditions evaluated by the EGS service include acute appendicitis, acute cholecystitis, intestinal obstruction, and gastrointestinal perforations [9]. A study conducted in Turkey reported that acute cholecystitis and acute appendicitis together accounted for approximately 68.81% of the surgeries performed by EGS services [10]. Furthermore, prior literature highlights persistent challenges in the regionalization of EGS services, attributed to the absence of standardized transfer criteria. This lack of uniformity contributes to unnecessary patient transfers, increased healthcare costs, and added strain on tertiary care resources [11,12].

To fill such gaps, the American Association for the Surgery of Trauma (AAST) created the Acute Care Surgery (ACS) model, prioritizing the multidisciplinary cooperation among surgeons, nurses, residents, and other healthcare providers available 24/7 to correctly manage emergency surgical cases [13,14]. Research shows that using the ACS model significantly decreases consultation times, hospital length of stay, morbidity, and even mortality, improving outcomes and the utilization of healthcare resources [6]. Despite these benefits, the literature is unfortunately still limited regarding the effectiveness, obstacles, and opportunities related to emergency general surgery consultations and transfers within Saudi Arabia’s healthcare system.

Studying the Saudi ACS model provides insights for other middle-income countries and tertiary centers where subspecialty coverage is limited, highlighting how integrated ACS services can optimize emergency surgical care.

This study aims to identify the general surgery subspecialties most frequently consulted by the emergency department at King Abdulaziz Medical City (KAMC).

## 2. Materials and Methods

This retrospective descriptive study was conducted at KAMC, under the Ministry of National Guard Health Affairs (MNGHA) in Riyadh, between September 2022 and November 2023. The study period (September 2022 to November 2023) was selected to provide the most recent consecutive 14-month interval with complete data availability, covering both high- and low-volume months and thereby representing a typical clinical year. The objective was to examine patterns of emergency general surgery (EGS) consultations and transfers of care, and to identify the general surgery subspecialties most frequently consulted by the emergency department. All consecutive patients admitted from the ED to the general surgery department were included. Referrals were identified exclusively through the BestCare electronic consultation system, which logs all ED-to-surgery consultations at our institution At KAMC, general surgeons provide care for pediatric surgical emergencies in the absence of a dedicated pediatric surgery service. Data were collected retrospectively through the BestCare System, which documented patients’ demographic characteristics, chief complaints, admission dates, admitting subspecialties, reasons for consultation, lengths of hospital stay, and discharge outcomes. A total of 384 patients met the inclusion criteria and were analyzed. Length of stay data were missing for 107 patients due to transfers or incomplete discharge documentation. Analyses were performed on available cases only, without imputation.

Statistical analyses were performed using RStudio software (R version 4.3.1). Categorical variables were summarized as frequencies and percentages, while continuous variables were expressed as medians with interquartile ranges (IQRs). Comparisons between categorical variables were conducted using Fisher’s exact test due to its suitability for small sample sizes and categorical data. A *p*-value of <0.05 was considered statistically significant. Data visualization techniques were used to illustrate key findings and highlight trends in consultation patterns. All statistical procedures adhered to best practices in medical research to ensure robust and reliable results. Given the exploratory nature of the study, no formal adjustment for multiple comparisons was performed. Findings should be interpreted as hypothesis-generating.

This study was approved by the Institutional Review Board at King Abdullah International Medical Research Center, Riyadh, Saudi Arabia (IRB Number: 2647/23). As this was a retrospective clinical audit involving de-identified data, the requirement for informed consent was waived by the ethics committee in accordance with institutional guidelines.

## 3. Results

### 3.1. Demographic and Clinical Characteristics of Patients

The study included 384 patients admitted to general surgery from the emergency department. Of these, 204 (53.1%) were male, and 180 (46.9%) were female. A total of 112 (29.2%) patients were aged 30 to <45 years, followed by 98 (25.5%) under 30 years. Diabetes and hypertension were the most common comorbidities, affecting 94 (24.5%) and 90 (23.4%) of patients, respectively (Table 1).

#### Description of Chief Complaints and Surgical Procedures

**Figure 1:**
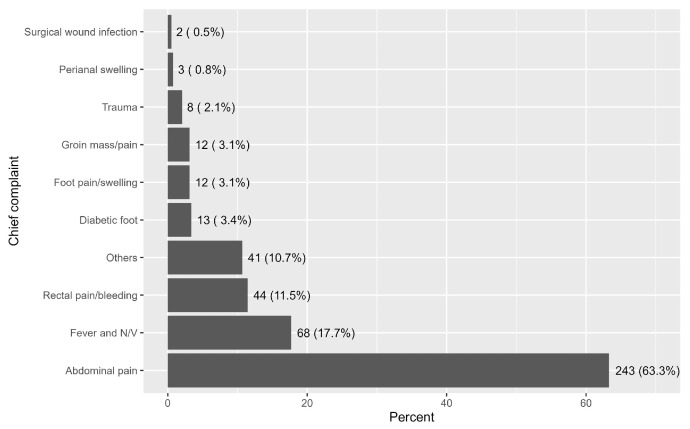
Description of patients’ chief complaints.

As depicted in Figure 1, the most common complaint was abdominal pain, reported by 243 patients (63.3%). This was followed by fever and nausea/vomiting in 68 patients (17.7%), and rectal pain/bleeding in 44 patients (11.5%, Figure 1).

### 3.2. Description of Surgeries Performed to Patients

The most frequently performed surgical procedure was laparoscopic cholecystectomy, performed in 123 patients (32.0%), followed by laparoscopic appendicectomy in 57 patients (14.8%), and anorectal examination in 45 patients (11.8%). The median length of stay was 3.0 days (IQR, 2.0 to 8.0, Table 2). Overall, 210 of 384 admitted patients (54.7%) underwent surgical intervention, representing the operative yield of the cohort.

### 3.3. Characteristics of Subspecialties and Referral Sites

Acute care was the most frequently consulted general surgery subspecialty, accounting for 231 patients (61.8%) of consultations, followed by colorectal, 86 (23.0%), and upper gastrointestinal (GI), 40 (10.7%). 

The overwhelming majority of referrals, 355 (98.9%), were from the emergency department to acute care surgery (Table 3).

### 3.4. Statistical Differences Based on Subspecialties

There was a significant difference in gender distribution among subspecialties (*p* = 0.049), with male patients more frequently consulting colorectal 56 (65.1%) and acute care 119 (51.5%). Liver disease was significantly associated with the referral to upper GI specialty (*p* = 0.008). Significant associations were also found between malignancy and subspecialty (*p* = 0.008), with colorectal patients exhibiting the highest proportion of malignancy, 12 (14.0%). Additionally, chief complaints such as abdominal pain (*p* < 0.001), fever and nausea/vomiting (*p* < 0.001), and rectal pain/bleeding (*p* < 0.001) were significantly associated with specific subspecialties (Table 4).

### 3.5. Statistical Differences Based on Referral Sites

Fever, nausea, and vomiting were significantly more common in patients referred to general surgery outpatient clinics compared to those referred to acute care (75.0% vs. 18.0%, *p* = 0.022). No other significant differences were observed between referral sites in terms of patient demographics or clinical characteristics (Table 5).

## 4. Discussion

Abdominal pain was the most common presenting complaint, reported in 243 patients (63.3%). This pattern likely reflects both the catchment area of King Abdulaziz Medical City (KAMC) and the centralized nature of its emergency general surgery (EGS) services, which receive a higher proportion of complex referrals than community hospitals. Standardized triage and rapid diagnostic pathways for abdominal pain are therefore particularly relevant for tertiary centers to maintain efficient patient flow and prevent emergency department (ED) overcrowding.

Laparoscopic cholecystectomy (32%) and laparoscopic appendectomy (14.8%) were the most frequent procedures. These findings correspond with local and international data [8,15], identifying appendicitis, cholecystitis, and bowel obstruction as the leading indications for emergency surgery. The predominance of laparoscopic approaches underscores the maturity of minimally invasive surgery (MIS) practices at our institution and supports the feasibility of wider MIS adoption across Saudi Arabia when appropriate training and infrastructure are in place. Our results are also consistent with multicenter national data [16], which reported comparable procedural distributions. However, the higher laparoscopic rate and shorter median hospital stay in our study (3 days vs. 4–5 days) likely reflect the advantages of a dedicated ACS model and enhanced MIS capability.

In contrast with other studies [17], found that although ACS implementation in a Canadian academic hospital reduced the mean time to surgery (221 → 192 min) and decreased after-hours operations (72.6% → 60.0%), it did not shorten length of stay (LOS) for appendectomy or cholecystectomy and even increased LOS for bowel obstruction cases (8 → 12 days). This likely reflects early-phase ACS adoption and differing patient demographics, whereas our mature model achieved a shorter overall stay (median 3 days)—highlighting the potential efficiency gains of a fully established ACS system.

The predominance of acute care surgery consultations (61.8%) aligns with previous reports indicating that ACS teams are the main pathway for emergency surgical admissions [13,14]. Compared with the ACS model described in [18], which managed 4100 emergency surgical admissions through a unified service combining trauma, orthopedics, and general surgery—our results demonstrate that a focused, subspecialty-based ACS framework can achieve superior efficiency. Their study reported an average postoperative length of stay of 11.7 ± 9.5 days for younger and 13.9 ± 9.1 days for older patients; our institution achieved a median hospital stay of only 3 days, reflecting the effectiveness of dedicated subspecialty triage and consultant-driven decision pathways in expediting care and discharge.

Similarly, Altıner et al. [19] reported that among 1513 general-surgery consultations in a Turkish tertiary hospital, only 79 (5.2%) resulted in operative intervention, indicating a substantial non-operative workload under a traditional consult-based model. In contrast, our ACS system achieved a much higher operative yield, with approximately 210 of 384 admissions (54.7%) undergoing surgery, including 123 laparoscopic cholecystectomies and 57 appendectomies. These findings underscore the efficiency of structured triage, continuous surgical coverage, and centralized consultant oversight characteristic of mature ACS frameworks.

The acute care surgery (ACS) model described by another international article [20] in the United States operates as a fully centralized, continuously staffed service that receives and manages all emergency surgical consultations around the clock. By comparison, the KAMC model functions through electronic triage via the BestCare system, where consultations are distributed to subspecialties according to presentation and complexity. This contrast underscores different stages of ACS development—Saudi tertiary centers emphasizing digital coordination and subspecialty collaboration.

Our observation that most surgical referrals originated from the emergency department and were directed to the ACS service reflects a parallel pattern within the Saudi healthcare system [21]. Although the referral sources differ—originating from the Emergency Department in our study and from primary care in the analysis by Al-Qahtani and Imtiaz—both findings highlight a similar national trend. They analyzed 1100 primary-care referral letters from a military hospital and found that surgical specialties accounted for 51.7% of all referrals. This demonstrates a longstanding reliance on secondary and tertiary surgical services in Saudi Arabia. Such a pattern suggests that the current centralization of emergency surgical care within ACS models represents a structured evolution of these earlier referral dynamics.

The overwhelming proportion of ED referrals in our cohort (98.9%) reinforces the emergency department’s central role as the primary gateway for surgical care in tertiary centers. In contrast, another local study reported that establishing urgent-care clinics (UCCs) reduced overall ED visits by 17.2% in some regions and decreased low-urgency presentations from 21.4% to 9.0%, highlighting their effectiveness in diverting non-critical cases and improving patient flow [22]. Implementing similar intermediate-care models within tertiary hospitals could help mitigate ED congestion and indirectly shorten consultation-to-decision intervals for surgical cases, thereby enhancing the responsiveness of emergency surgical services.

Overall, these findings have several system-level implications. The high rate of abdominal-pain presentations supports the need for dedicated diagnostic algorithms and observation units to expedite assessment. The substantial proportion of laparoscopic procedures emphasizes the importance of sustaining MIS expertise and infrastructure in emergency settings. Finally, the predominance of ACS consultations highlights the value of structured emergency-surgery models in tertiary care centers and suggests that wider implementation across Saudi Arabia could enhance timely access to surgical care. Future research should focus on outcome benchmarking, cost-effectiveness, and workforce planning to further optimize emergency surgical service organization.

## 5. Limitations

This study has several limitations. First, its retrospective design depended on the accuracy and completeness of electronic medical records, resulting in some missing data, including length of stay for 12% of cases. Second, as a single-center study conducted in a tertiary care facility, the findings may not reflect practices or referral patterns in secondary or rural hospitals. Third, the analysis focused only on initial consultation data without capturing final diagnoses, management outcomes, or long-term follow-up, limiting assessment of referral appropriateness and patient outcomes. Lastly, the study period overlapped with the COVID-19 pandemic, which may have influenced case volume and presentation patterns. Despite these limitations, the data provide meaningful insights into emergency surgical trends within an acute care surgery model.

## 6. Conclusions

This retrospective study found that abdominal pain was the most frequent presenting complaint in the emergency department, and laparoscopic cholecystectomy and appendectomy were the most commonly performed surgeries. The predominance of acute care surgery referrals reflects the structure of the Saudi ACS model, which centralizes emergency general surgery, trauma, and critical care under one service. This organizational approach facilitates timely access to surgical care and enables high uptake of minimally invasive techniques. Beyond documenting local patterns, our findings highlight how the Saudi ACS model may offer lessons for tertiary hospitals and health systems internationally, particularly in regions where subspecialty availability is limited. Broader adoption of integrated ACS frameworks could improve efficiency, expand access to minimally invasive surgery, and enhance outcomes in emergency surgical care.

## Figures and Tables

**Table 1:** Demographic and clinical characteristics of patients.

Characteristic	Description
Age (years)	-
<30	98 (25.5%)
30 to <45	112 (29.2%)
45 to <60	86 (22.4%)
60 or more	88 (22.9%)
Gender	-
Male	204 (53.1%)
Female	180 (46.9%)
Comorbidities	-
Diabetes	94 (24.5%)
Hypertension	90 (23.4%)
Cardiovascular disease	37 (9.6%)
Chronic obstructive pulmonary disease	1 (0.3%)
Chronic kidney disease	16 (4.2%)
Liver Disease	6 (1.6%)
Malignancy	23 (6.0%)
Irritable bowel syndrome	5 (1.3%)
*n* (%)	

**Table 2:** Description of surgeries performed to patients.

Characteristic	Description
Surgical intervention	-
Amputation	11 (2.9%)
Anorectal examination	45 (11.7%)
Colonic procedure	16 (4.2%)
Diagnostic laparoscopy	22 (5.7%)
Exploratory laparotomy	20 (5.2%)
Gastrectomy	3 (0.8%)
Hernia repair	27 (7.0%)
Incision and drainage	9 (2.3%)
Laparoscopic appendicectomy	57 (14.8%)
Laparoscopic cholecystectomy	123 (32.0%)
Laparoscopic cholecystectomy proceeding to open cholecystectomy	5 (1.3%)
Pilonidal sinus	7 (1.8%)
Soft tissue excision	17 (4.4%)
Others	11 (2.9%)
Length of stay (days)	3.0 (2.0–8.0)
*n* (%); Median (IQR) The variable had 107 missing records	

**Table 3:** Characteristics of subspecialties and referral sites.

Characteristic	Description
Subspecialty	-
Colorectal	86 (23.0%)
Acute care	231 (61.8%)
Upper GI	40 (10.7%)
Oncology	12 (3.2%)
Endocrine	3 (0.8%)
Vascular	2 (0.5%)
Referral site	-
Acute care (From ER to general surgery)	355 (98.9%)

**Table 4:** Statistical differences based on subspecialties in terms of patients’ demographic and clinical characteristics and chief complaints. *p*-value is considered significant (*p* < 0.05, 0.001).

Characteristic	Colorectal N = 86	Acute Care N = 231	Upper GI N = 40	Oncology N = 12	Endocrine N = 3	Vascular N = 2	*p*-Value
Age (years)	-	-	-	-	-	-	0.069
<30	11 (12.8%)	70 (30.3%)	11 (27.5%)	2 (16.7%)	1 (33.3%)	0 (0.0%)	-
30 to <45	30 (34.9%)	62 (26.8%)	12 (30.0%)	5 (41.7%)	0 (0.0%)	0 (0.0%)	-
45 to <60	23 (26.7%)	48 (20.8%)	8 (20.0%)	4 (33.3%)	2 (66.7%)	0 (0.0%)	-
60 or more	22 (25.6%)	51 (22.1%)	9 (22.5%)	1 (8.3%)	0 (0.0%)	2 (100.0%)	-
Gender	-	-	-	-	-	-	0.049
Male	56 (65.1%)	119 (51.5%)	15 (37.5%)	6 (50.0%)	1 (33.3%)	1 (50.0%)	-
Female	30 (34.9%)	112 (48.5%)	25 (62.5%)	6 (50.0%)	2 (66.7%)	1 (50.0%)	-
Comorbidities	-	-	-	-	-	-	-
DM	23 (26.7%)	54 (23.4%)	9 (22.5%)	3 (25.0%)	1 (33.3%)	1 (50.0%)	0.830
HTN	23 (26.7%)	53 (22.9%)	7 (17.5%)	1 (8.3%)	1 (33.3%)	1 (50.0%)	0.467
CVD	9 (10.5%)	22 (9.5%)	2 (5.0%)	2 (16.7%)	1 (33.3%)	0 (0.0%)	0.401
COPD	0 (0.0%)	1 (0.4%)	0 (0.0%)	0 (0.0%)	0 (0.0%)	0 (0.0%)	>0.999
CKD	4 (4.7%)	7 (3.0%)	2 (5.0%)	0 (0.0%)	0 (0.0%)	1 (50.0%)	0.175
Liver Disease	1 (1.2%)	1 (0.4%)	3 (7.5%)	0 (0.0%)	0 (0.0%)	0 (0.0%)	0.049
Malignancy	12 (14.0%)	6 (2.6%)	2 (5.0%)	1 (8.3%)	0 (0.0%)	0 (0.0%)	0.008
IBD	3 (3.5%)	1 (0.4%)	1 (2.5%)	0 (0.0%)	0 (0.0%)	0 (0.0%)	0.143
Abdominal pain	30 (36.6%)	167 (74.9%)	31 (79.5%)	8 (88.9%)	0 (0.0%)	0 (0.0%)	<0.001
Fever and N/V	5 (6.1%)	50 (22.4%)	6 (15.4%)	5 (55.6%)	0 (0.0%)	0 (0.0%)	<0.001
Others	10 (12.2%)	16 (7.2%)	7 (17.9%)	1 (11.1%)	3 (100.0%)	0 (0.0%)	<0.001
Rectal pain/bleeding	39 (47.6%)	4 (1.8%)	1 (2.6%)	0 (0.0%)	0 (0.0%)	0 (0.0%)	<0.001
Diabetic foot	0 (0.0%)	13 (5.8%)	0 (0.0%)	0 (0.0%)	0 (0.0%)	0 (0.0%)	0.119
Foot pain/swelling	3 (3.7%)	7 (3.1%)	1 (2.6%)	0 (0.0%)	0 (0.0%)	1 (100.0%)	0.131
Groin mass/pain	1 (1.2%)	10 (4.5%)	0 (0.0%)	0 (0.0%)	0 (0.0%)	0 (0.0%)	0.500
Perianal swelling	2 (2.4%)	1 (0.4%)	0 (0.0%)	0 (0.0%)	0 (0.0%)	0 (0.0%)	0.359
Surgical wound infection	1 (1.2%)	1 (0.4%)	0 (0.0%)	0 (0.0%)	0 (0.0%)	0 (0.0%)	0.610
Trauma	0 (0.0%)	7 (3.1%)	0 (0.0%)	0 (0.0%)	0 (0.0%)	0 (0.0%)	0.405
*n* (%)							
Fisher’s exact test							

**Table 5:** The outpatient referral group consisted of only four patients, limiting the statistical reliability of comparisons. These results should be interpreted descriptively rather than conclusively.

Characteristic	Acute Care N = 355	General Surgery Outpatient Clinic N = 4	*p*-Value
Age (years)	-	-	0.083
<30	96 (27.0%)	0 (0.0%)	-
30 to <45	104 (29.3%)	1 (25.0%)	-
45 to <60	78 (22.0%)	0 (0.0%)	-
60 or more	77 (21.7%)	3 (75.0%)	-
Gender	-	-	>0.999
Male	189 (53.2%)	2 (50.0%)	-
Female	166 (46.8%)	2 (50.0%)	-
Comorbidities	-	-	-
DM	83 (23.4%)	2 (50.0%)	0.239
HTN	78 (22.0%)	2 (50.0%)	0.216
CVD	34 (9.6%)	0 (0.0%)	>0.999
COPD	1 (0.3%)	0 (0.0%)	>0.999
CKD	15 (4.2%)	0 (0.0%)	>0.999
Liver Disease	6 (1.7%)	0 (0.0%)	>0.999
Malignancy	21 (5.9%)	0 (0.0%)	>0.999
IBD	5 (1.4%)	0 (0.0%)	>0.999
Chief complaint	-	-	-
Abdominal pain	225 (66.4%)	3 (75.0%)	>0.999
Fever and N/V	61 (18.0%)	3 (75.0%)	0.022
Others	40 (11.8%)	0 (0.0%)	>0.999
Rectal pain/bleeding	41 (12.1%)	1 (25.0%)	0.408
Diabetic foot	13 (3.8%)	0 (0.0%)	>0.999
Foot pain/swelling	8 (2.4%)	0 (0.0%)	>0.999
Groin mass/pain	10 (2.9%)	0 (0.0%)	>0.999
Perianal swelling	3 (0.9%)	0 (0.0%)	>0.999
Surgical wound infection	1 (0.3%)	0 (0.0%)	>0.999
Trauma	8 (2.4%)	0 (0.0%)	>0.999
*n* (%)			
Fisher’s exact test			

## Data Availability

The original contributions presented in this study are included in the article. Further inquiries can be directed to the corresponding author(s).

## Abbreviations

The following abbreviations are used in this manuscript:

ACS	Acute Care Surgery
AAST	American Association for the Surgery of Trauma
EGS	Emergency General Surgery
ED	Emergency Department
GI	Gastrointestinal
GS	General Surgery
IQR	Interquartile Range
IRB	Institutional Review Board
KAMC	King Abdulaziz Medical City
MNGHA	Ministry of National Guard Health Affairs
RR	Relative Risk
RStudio	R Statistical Computing Software
